# Effective Interaction Strength in Simulations of Liquid Mixtures: A Configuration-Dependent Species-Specific Measure of Interaction Enthalpies

**DOI:** 10.1021/acs.jpcb.5c03339

**Published:** 2025-07-21

**Authors:** Anna Luisa Upterworth, Daniel Sebastiani

**Affiliations:** Institute of Chemistry, Martin Luther University Halle-Wittenberg, Halle 06120, Germany

## Abstract

The thermodynamics of mixing and demixing of molecular liquids poses a perpetual challenge for atomistic simulations. We provide a computational tool, the effective interaction strength, which can be used to quantify the enthalpic contribution of individual species-dependent interactions. The effective interaction strength is configuration-dependent and thus reflects how the actual simulated state (i.e., mixed or phase separated) affects the average particle interactions. Beyond that, the effective interaction strength can be utilized to gain atomistic insight into complex phenomena, including the fundamental nature of the philicity of a compound and the contributions of individual functional groups to this philicity.

## Graphical abstract

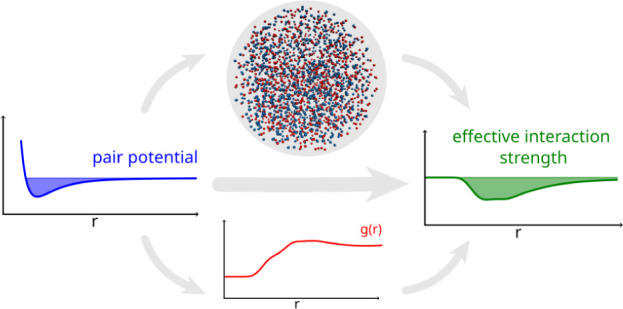

## Introduction

It is the intra- and intermolecular interactions, that determine the state of chemical systems, such as whether the components of a liquid mixture will mix or separate into separate phases. Molecular dynamics (MD) simulations provide a powerful tool for understanding of the underlying microscopic processes that lead to a specific macroscopic state.^1,2^ Traditionally, there are two opposing approaches to simulate the interactions. In highly accurate *ab initio* molecular dynamics (AIMD) simulations, the electronic structure is computed at each time step,^3^ mostly by density functional theory (DFT).^4,5^ In contrast, in much faster but less accurate classical molecular dynamics simulations, the total potential energy as a function of particle coordinates is obtained from empirical terms for bonded and nonbonded interactions.^6,7^ With the goal of combining the advantages of these two approaches, i.e., the quantum-chemical accuracy of AIMD and the simulation speed of force fields^2,8^ machine learning approaches have also attracted attention in recent years. However, due to their higher complexity large quantum-chemical data sets are required for training^2,5^ and their computational speed is still one to 2 orders of magnitude slower compared to classical force fields.^5^ In addition, the high computational cost limits the applicability of AIMD both in terms of system size and simulation time scale, which is why empirical force field MD often remains the method of choice for simulations of liquids and large biomolecules.^5,9^

While the exact functional form varies between different approaches, many nonreactive force fields used in simulations of molecular liquids model the bonded contribution as a combination of potentials for bond stretching, angle bending as well as rotations around single bonds.^6–8^ Popular examples of this simple general functional form are the OPLS-AA,^10,11^ AMBER,^12^ CHARMM,^9,13^ GROMOS96,^14^ and TraPPE,^15^ force fields. The nonbonded, longer range intermolecular interactions are usually accounted for by pairwise additive Coulomb and van der Waals interactions.^7^ A prominently used variant to describe the latter is the Lennard-Jones 12–6 potential (eq 1)^7,16^

1 $$U^{\mathrm{LJ}}\left(r\right)=4\varepsilon \left[{\left(\frac{\sigma }{r}\right)}^{12}-{\left(\frac{\sigma }{r}\right)}^{6}\right]$$

where *r* is the distance between the two interacting particles, and the energy and size parameters *ε* and σ are defined as the depth of the potential well and the point of zero crossing, respectively.

Considerable effort is put into finding potentials that accurately describe the actual behavior of the system. Effective interaction potentials are parametrized to include many-body effects in the pairwise interaction energies,^6^ which consequently differ from the true physical interaction. Strategies to obtain them from target structural data include inverse Monte Carlo^17–19^ iterative Boltzmann inversion^20,21^ or integral equation theory,^22^ for example via the inverse solution of reference interaction site model (RISM).^23^ However, finding the most accurate effective pair potentials is not what we are concerned with in this work. Rather, we want to quantify the state-dependent relevance of the interaction of a given species-pair. While the underlying fundamental pairwise interaction functions *U*_*ab*_(*r*) applied in a simulation are themselves configuration independent, their interplay determines the states sampled in the simulation trajectory. Thus, all properties computed as ensemble averages during postprocessing are configuration dependent. This includes both scalar properties such as the total energy, pressure, entropy, etc. as well as functions such as spatial (SDF) and radial distribution functions (RDF, *g*(*r*)). The latter represent the probability of finding an observed particle (atom or molecule) at a distance *r* from another reference particle and thereby characterize the local liquid structure. Assuming pairwise additivity of the forces, many thermodynamic properties can be calculated from RDFs.^6,24^ For the internal energy of the most simplistic one-component system of interacting point particles, this is shown in eq 2 ^25,26^

2 Utotal=Uideal+Uexcess=32NkBT+4πN22V∫0∞r2U(r)g(r)dr

where *N* is the number of particles, *V* the volume, and *U*(*r*) the pair potential. The factor 1/2 avoids double counting of interactions. Other thermodynamic properties such as compressibility, partial molar volumes, and derivatives of the chemical potential are accessible via the related Kirkwood-Buff integrals.^27,28^

The excess internal energy in eq 2, sometimes referred to as the configurational internal energy, measures the fraction of internal energy caused by interactions.^29^ For mixtures of *K* components, it takes the form

3 $$U^{\mathrm{excess}}=4\pi \frac{N_{a}N_{b}}{2V}\rho \overset{K}{\underset{a=1}{\sum }}\overset{K}{\underset{b=1}{\sum }}x_{a}x_{b}\int_{0}^{\infty }r^{2}U_{ab}\left(r\right)g_{ab}\left(r\right)dr$$

where *x*_*a*_ is the mole fraction of component *a* and *U*_*ab*_(*r*) is the pair potential between molecules *a* and *b*.^26^ *U* ^excess^ in eq 3 corresponds to the enthalpy of the system. Together with the configurational entropy it determines the free energy (*G*), which of course also depends on the configuration. The result of a converged simulation, regardless of whether molecular dynamics or Monte Carlo was used, is a trajectory with the average free energy ⟨Δ*G*⟩_traj_. Our goal is to decipher the species-wise contributions to the enthalpic part of ⟨Δ*G*⟩_traj_, i.e., ⟨Δ*H*⟩_traj_. For this reason, we suggest expressing the importance of each species as an easy-to-analyze quantity, i.e., the effective interaction strength.

Related concepts have already been explored by other authors, some of which we would like to mention here. Bahar and Jernigan calculated solvent-mediated effective contact potentials and effective self-contact potentials between protein residues from radial distribution functions.^30^ Mochizuki and Koga used Kirkwood-Buff theory to quantify the cononsolvency of methane in water–methanol mixtures by decomposing the excess chemical potential into contributions from effective solute-water and solute-methanol interactions.^31^ In their work, the preferential interaction with one solvent over the other is measured by the difference in Kirkwood-Buff integrals, which cover the effective solute–solvent interaction over the entire range of intermolecular distances.^31^ Recently, Adachi and Kawaguchi presented an approach to estimate the effective interaction strength of polypeptide chains based on second virial coefficients obtained from the potential of mean force, where the overall attraction or repulsion results from additive contributions of the pairwise interactions between single amino acids (monomers) or two adjacent amino acids (dimers).^32^ To predict the phase separation of two amino acid sequences (components), these authors introduce an effective interaction parameter as the difference between the intercomponent and intracomponent interaction strengths.^32^ All of these approaches, however, work at the molecular level or with coarse-grained models and are aimed at the description of the totality of all interactions. The desired distinction and comparison of the influence of the individual species–species interactions is not achieved in this way.

Another possibility is to differentiate according to the type of interaction. This has been done for example by Erlebach et al., who predicted the solubilities of several polymer–solvent combinations from Hansen solubility parameters within the framework of Flory–Huggins theory and analyzed the underlying interactions by splitting the cohesive energy density obtained from eq 3 with square-well intersegment potentials into electrostatic and van der Waals contributions.^33^ While this is useful and could at a later stage be combined with our descriptor of species-wise effective interaction strengths to better understand the underlying forces in physically more complex systems, for the scope of this work we will not make this further division into proportions of different interaction types.

It should be further noted, that the term effective interaction strength has already been used in other contexts. In the field of superconductors it measures the overall interaction between electron pairs with phonon-induced attractive and repulsive Coulomb contributions.^34,35^ Liu et al. compared the effective Coulomb interaction strength of lanthanide metals calculated with different first-principles approaches and analyzed the magnitudes of f-orbital localization and screening strength.^36^ Since the common feature of these concepts is that there are two competing forces, it seems reasonable to apply the term to molecular systems in which attractive and repulsive intermolecular interactions compete.

## Methods

A series of MD simulations was performed on a mixture of hexane–perfluorohexane at six temperatures between 200 and 300 K using the LAMMPS^37,38^ program package and the OPLS-AA^11^ (Optimized Potentials for Liquid Simulations—all atom) force field. The force field parameters were taken as published by the OPLS developers for alkanes^11^ and perfluorinated alkanes.^39^ The geometric mean rule was used except for the H–F interaction, for which special mixing rules proposed by Morgado et al.^40^ were applied. Pairwise Coulomb and Lennard-Jones interactions were calculated directly up to a cutoff distance of 8 Å. Longer range Coulomb interactions were computed by the pppm (particle–particle particle-mesh) solver.^41^ A time step of 1 fs was used. The cubic simulation box containing 250 hexane and 250 perfluorohexane molecules was prepared by PACKMOL.^42^ Prior to each MD simulation, the energy was minimized with stopping tolerances of 10^–4^ and 10^–6^ kcal/(molÅ) for the energy and force. A multistep equilibration protocol was then performed to determine the equilibrium density. Unless otherwise stated, temperature and pressure control were achieved by Nosé-Hoover thermostats^43–45^ with a coupling constant of 100 fs and barostats with a coupling constant of 2000 fs. Velocity initialization according to the Maxwell distribution at the simulation temperature was followed by 25 ps of initial equilibration in an NVE ensemble with direct temperature rescaling and 50 ps in an NVT ensemble. The ensemble was then changed to NpT. After 50 ps, a Langevin dynamics run with a coupling constant of 100 fs was performed for another 50 ps to dampen shock waves caused by the change in system size. The density was then determined by averaging the last 2.5 ns of the following longer (3.5 ns) run in the NpT ensemble. Afterward, the simulation box was resized according to the determined density, and the ensemble was changed back to NVT. After 25 ps, a second Langevin dynamics run was performed to dampen shock waves for 0.25 ns in the NVE ensemble before final equilibration for 1 ns in the NVT ensemble. Production runs were then performed for 10 ns in the NVT ensemble, with every 100th step written to the trajectory file.

For more information on final densities and box volumes at the different temperatures, see Table S1. TRAVIS^46,47^ was used to compute RDFs for all possible combinations of atom types according to eq 4. Every 100th trajectory frame was used for analysis, and the average was taken over the entirety of the production run.

4 $$g_{ab}\left(r\right)=\frac{V}{N_{a}N_{b}}\overset{N_{a}}{\underset{i=1}{\sum }}\overset{N_{b}}{\underset{j=i+1}{\sum }}\left\langle \delta (r-\right|{\mathrm{r⃗}}_{i}\left(t\right)-{\mathrm{r⃗}}_{j}\left(t\right)|)\rangle_{t}$$

Snapshots depicting atomic configurations were rendered from VMD^48^ using Tachyon,^49^ and all plots were generated using Matplotlib,^50^ a Python library. The effective interaction strengths were obtained according to eq 5 by numerical integration of *r* ^2^ *U*_*ab*_(*r*)*g*_*ab*_(*r*) employing the scipy.integrate.trapezoid method^51^ and subsequent multiplication with the respective prefactor. The integral values and prefactors used are given in Tables S2 and S3.

## Results and Discussion

In the expression for the excess internal energy of a mixture (eq 3), the atomistic pair interaction potential *U*_*ab*_(*r*) is weighted with the partial radial density distribution, and the total configurational internal energy is subsequently obtained by summing over all components. In this work, we propose to use the density weighted interaction potential (without the summation) as a descriptor for the *relevance of atomistic pairwise interactions*. Specifically, the effective interaction strength of a pairwise interaction between *a* and *b* is

5 $$U_{ab}^{\mathrm{eff}}=4\pi f_{ab}\frac{N_{a}N_{b}}{V}\int r^{2}U_{ab}\left(r\right)g_{ab}\left(r\right)dr$$

where the factor $f_{ab}=\frac{1}{2}$ for *a* = *b* and *f*_*ab*_ = 1 for *a* ≠ *b* ensures correct counting of the interaction pairs.

In order to illustrate the principle of operation of this descriptor $U_{ab}^{\mathrm{eff}}$ , let us consider a binary mixture of uncharged Lennard-Jones point particles of types 1 and 2. The instantaneous state of such a mixture can be fully phase-separated, perfectly mixed, and, naturally, every state in between. These two extreme cases are illustrated in Figure 1, where particles of species 1 are colored in blue and those of species 2 are red. While the entropic aspect of this phase state can be determined from a recently proposed expression for the configurational entropy of mixing,^52^ the enthalpic counterpart can be described at a species-specific level by using eq 5. In the case of two completely separated pure phases, the homointeractions between particles of the same kind are fully dominant over heterointeractions between particles of different types. Consequently, the effective interaction strengths $U_{11}^{\mathrm{eff}}$ and $U_{22}^{\mathrm{eff}}$ reach their maximum in this state, whereas the effective strength $U_{12}^{\mathrm{eff}}$ is effectively zero. The converse is true in the configuration corresponding to a perfectly mixed state. Here, $U_{12}^{\mathrm{eff}}$ attains its maximum value, while $U_{11}^{\mathrm{eff}}$ and $U_{22}^{\mathrm{eff}}$ are at their minimum for this specific system composition. It is noteworthy that the particle-level interaction potentials *U*_*ab*_(*r*) are the same in both states.

**Figure 1:**
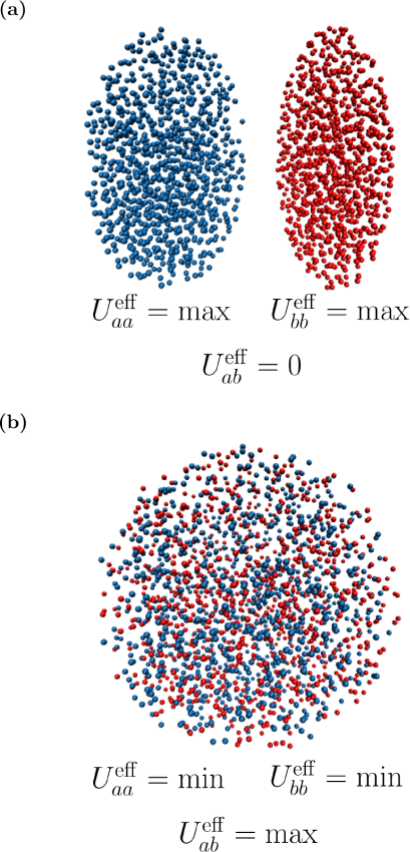
Schematic illustration of eq 5 for a system of two types of Lennard-Jones point particles (blue and red). Extreme cases include (a) complete phase separation and (b) random mixture.

As one transitions from the description of interactions between Lennard-Jones point particles to that of chemical molecules, the model becomes more complex. Interacting compounds and actually interacting particles are no longer equivalent. Instead, we are looking at a mixture of molecules (compounds) that each consist of several atoms. Within the framework of an pairwise additive force field, interactions are considered at the atomic level between different atom types. In this context, the application of eq 5 as a descriptor of effective interaction strengths remains valid without adaptation. However, the concept of species becomes more intricate due to the fact that a specific atomic species (e.g., a carbon atom) can be found in multiple different molecules, as well as in various variants within a single molecule. Therefore, the indices *a* and *b* in eq 5 correspond now to a given species of a given type of molecule. For instance, a water–methanol mixture contains three hydrogen, two oxygen, and one carbon species which give rise to 21 distinct effective interaction strength values (for a given thermodynamic state). Another important detail is that any precise description of interactions in real molecules necessitates the incorporation of Coulomb forces, which have to be considered in addition to the van der Waals forces modeled via Lennard-Jones potentials in the examples above. Since the sheer number of effective interaction energy values grows quickly with the complexity of the molecular components of a mixture, a natural (optional) simplification is to consolidate all atomic species belonging to a given molecule. This yields a molecular effective interaction energy value according to eq 6. The relevance of individual interactions can be accessed by comparison of the effective interaction strengths $U_{ab}^{\mathrm{eff}}$ for different atoms *a* and *b*.

6 $$U_{AB}^{\mathrm{eff}}=\underset{a\in A}{\sum }\underset{b\in B}{\sum }U_{ab}^{\mathrm{eff}}$$

The extension of this methodology to more complex mixtures is straightforward. The addition of a third molecule, *C*, constituted by atoms *c* ∈ *C* entails, for instance, the inclusion of pairwise interaction potentials *U*_*ac*_ and *U*_*bc*_. While of course all interactions contribute to the total energy, it is still possible to consider the contributions of the interactions between two types of molecules individually, i.e., to assess $U_{AB}^{\mathrm{eff}}$ , $U_{AC}^{\mathrm{eff}}$ , and $U_{BC}^{\mathrm{eff}}$ . Under the assumption that *C* partially mixes with both *A* and *B*, the *A* and *B* molecules will be diluted, and the densities ρ_*aa*_, ρ_*bb*_, and ρ_*ab*_ will be lower than that of the binary mixture. It can be deduced that the effective interaction energies $U_{AA}^{\mathrm{eff}}$ , $U_{BB}^{\mathrm{eff}}$ , and $U_{AB}^{\mathrm{eff}}$ will also be reduced.

To illustrate the applicability of our tool for simulation analysis, we performed MD simulations of a simple binary hexane–perfluorohexane mixture at varying temperatures, resulting in different configurational states. The OPLS-AA force field^11^ was used, and four different atom types were considered: hydrogen atoms (H), carbon atoms in hexane (C_H_), fluorine atoms (F), and carbon atoms in perfluorohexane (C_F_). Both van der Waals and Coulomb interactions are implemented in the OPLS functional form^11^ and determine the configurational state that is sampled in the MD trajectory. In this analysis, however, the focus is on the effect of the Lennard-Jones term, since dispersion forces dominate over electrostatic interactions in the specific system used.^53^ Effective interaction strengths $U_{ab}^{\mathrm{eff}}$ can be computed for all possible pairwise interactions H–H, H–C_H_, C_H_–C_H_, F–F, F–C_F_, C_F_–C_F_, H–F, H–C_F_, F–C_H_, and C_H_–C_F_. Taking the sums according to eq 7 yields the total effective interaction energies of the homomolecular interactions $U_{C_{6}H_{14}-C_{6}H_{14}}^{\mathrm{eff}}$ and $U_{C_{6}F_{14}-C_{6}F_{14}}^{\mathrm{eff}}$ as well as the heteromolecular interaction $U_{C_{6}H_{14}-C_{6}F_{14}}^{\mathrm{eff}}$ , which are equivalent to $U_{AA}^{\mathrm{eff}}$ , $U_{BB}^{\mathrm{eff}}$ , and $U_{AB}^{\mathrm{eff}}$ as previously used.

7 $$\begin{array}{l} U_{C_{6}H_{14}-C_{6}H_{14}}^{\mathrm{eff}}=U_{HH}^{\mathrm{eff}}+U_{HC_{H}}^{\mathrm{eff}}+U_{C_{H}C_{H}}^{\mathrm{eff}}\\ U_{C_{6}F_{14}-C_{6}F_{14}}^{\mathrm{eff}}=U_{FF}^{\mathrm{eff}}+U_{FC_{H}}^{\mathrm{eff}}+U_{C_{H}C_{H}}^{\mathrm{eff}}\\ U_{C_{6}H_{14}-C_{6}F_{14}}^{\mathrm{eff}}=U_{HF}^{\mathrm{eff}}+U_{HC_{H}}^{\mathrm{eff}}+U_{FC_{H}}^{\mathrm{eff}}+U_{C_{H}C_{H}}^{\mathrm{eff}} \end{array}$$

Finally, by calculating the difference between the sums of the effective interaction energies of hetero- and homomolecular interactions (eq 8), an effective energy of mixing Δ_mix_ *U* ^eff^ can be obtained. Note that this quantity is not equal to the total effective interaction energy *U* ^excess^, i.e., the sum of all individual effective interaction strengths, but rather has the character of a mixing enthalpy.

8 $$\Delta_{\mathrm{mix}}U^{\mathrm{eff}}=U_{C_{6}H_{14}-C_{6}F_{14}}^{\mathrm{eff}}-\left(U_{C_{6}H_{14}-C_{6}H_{14}}^{\mathrm{eff}}+U_{C_{6}F_{14}-C_{6}F_{14}}^{\mathrm{eff}}\right)$$

We have performed a systematic temperature variation to describe the mixing of hexane and perfluorohexane between 200 and 300 K. A snapshot of the MD trajectory simulated at 300 K is shown in Figure 2a.

**Figure 2:**
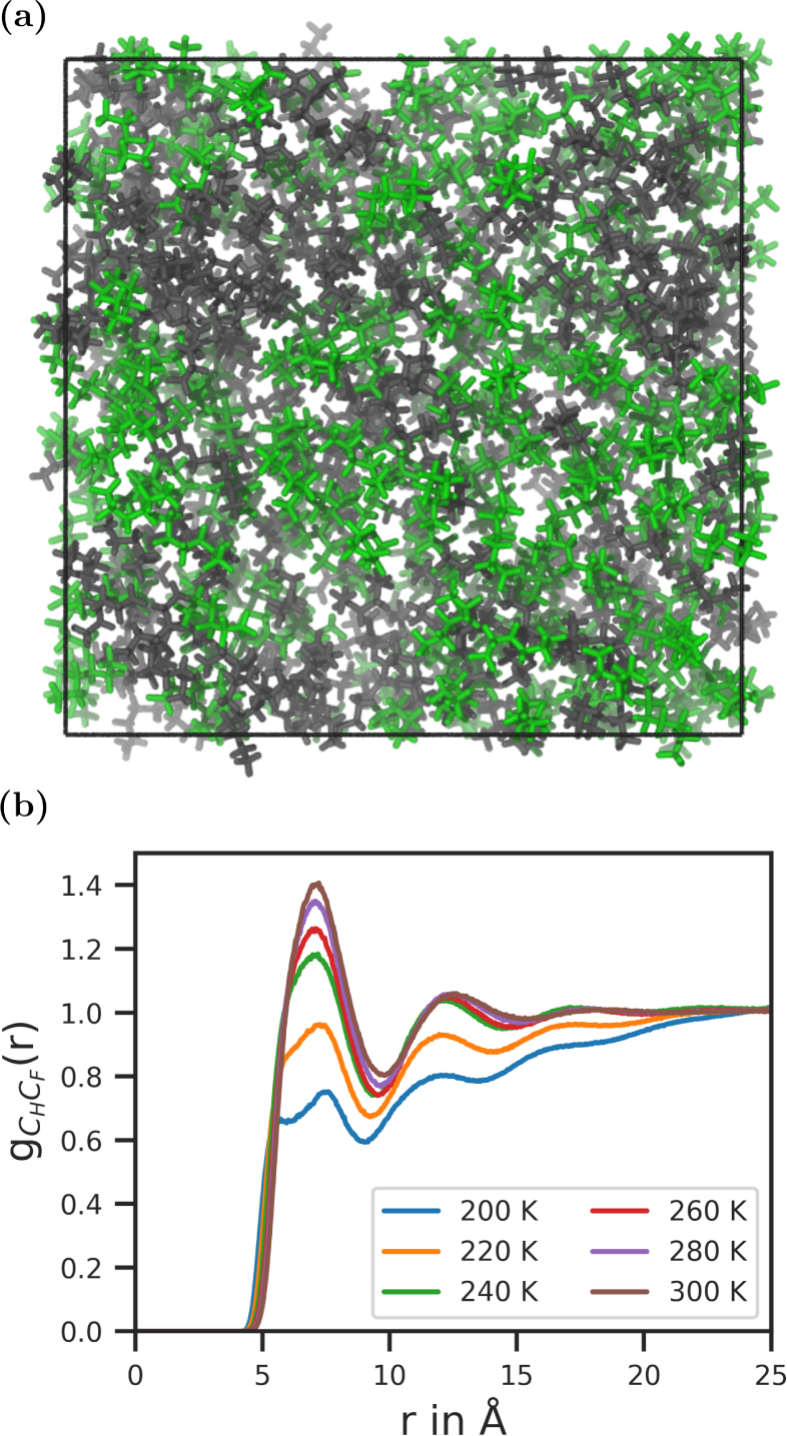
(a) Snapshot of the MD trajectory at 300 K. Hexane molecules are colored in gray and perfluorohexane molecules are colored in green. (b) Evolution of the radial distribution function between carbon atoms of hexane and perfluorohexane molecules with temperature.

It is evident, that the hexane (gray) and perfluorohexane (green) molecules are partially mixed, but their densities are spatially heterogeneous. To some extent, the degree of mixing can be determined from the height of the first peak in RDFs. Figure 2b shows the RDF between the carbon atoms of hexane (C_H_) and perfluorohexane (C_F_) at all simulated temperatures. The peak height increases with temperature, representing an increase in the degree of mixing. Nevertheless, it is impossible to predict the configurational states by knowing the temperature and the interaction potential parameters *ε*_*ab*_ and σ_*ab*_ without actually performing the simulation. The reason for this is that there is no understanding of the importance of single interactions in an observed state. Exactly this insight can be provided by the effective interaction strength, which is shown below.

Figure 3 shows the elementary functions of the calculation of the effective interaction strength for the example of the F–F interaction at 300 K. The 12–6 potential for the interaction of two fluorine atoms is plotted as the blue line in Figure 3a. The minimum is located at *r* = 3.3 Å and has a depth of *ε*_*FF*_ = 0.053 kcal/mol.^39^ Naturally, the fact that the potential is most attractive in its well around this minimum makes it tempting to believe that this distance region up to around 4 Å is the most important. Contrary to this assumption, the RDF between fluorine atoms obtained from the MD simulation at 300 K reveals that F–F distances between 2.5–4 Å (Figure 3b) are less common than larger distances where there is only weak attraction. The importance of the long-range interactions becomes even more apparent when looking at the integrand of eq 5 plotted as the green line in Figure 3c. The first positive peak at distances 2.5 < *r* < 3.3 Å corresponds to repulsion, before the attractive regime with negative energies is reached at larger distances. This repulsive contribution is first counterbalanced by the short-range attractive contribution up to about 4 Å, the distance range intuitively thought to have the largest weight when the effective interaction strength is calculated by integration. Therefore, the attractiveness of the F–F interaction with an effective strength of −243 kcal/mol at 300 K is mainly determined by the long-range interactions, despite the fact that the fluorine atoms are the outermost atoms in alkyl chains and therefore always are in direct contact with each other.

**Figure 3:**
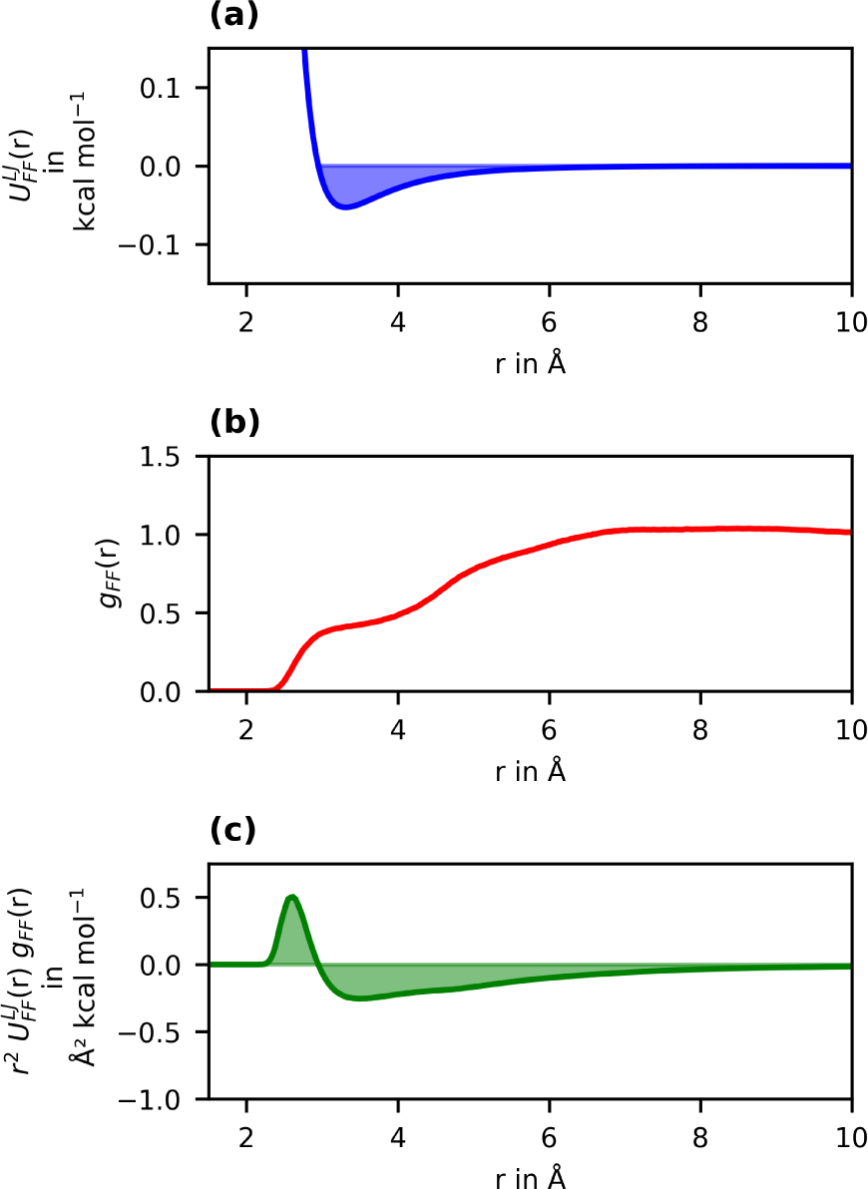
Elementary functions of the calculation of the effective interaction strength for the interaction between fluorine atoms. (a) F–F interaction potential according to the OPLS-AA force field (12–6 Lennard-Jones, *ε*_*FF*_ = 0.053 kcal/mol, σ_*FF*_ = 2.95 Å).^39^ (b) Radial distribution function between fluorine atoms obtained from an MD simulation of a hexane–perfluorohexane mixture at 300 K. (c) Effective interaction potential *r* ^2^ *U*_*ab*_(*r*)*g*_*ab*_(*r*) as the integrand of eq 5.

For the nine other pairwise interactions, we also obtained overall attractive effective interaction strengths (compare Figures 4 and S1–S8). The exact value is determined by the magnitudes of the repulsive and attractive regions, which in turn depend on where the RDF peaks are located relative to the minimum of the corresponding pair potential. In some cases, including the F–F interaction discussed above, there are some pair distances that lie in the repulsive energy region and thus contribute the positive part to the integrand of eq 5. These are a result of the restriction of the freedom of arrangement of individual atoms by the chemical bonds in the molecule in combination with the complex interplay of all interactions in the system. Other atom types naturally have larger distances between each other, so that the positive contribution to *r* ^2^ *U*_*ab*_(*r*)*g*_*ab*_(*r*) is smaller or even zero. This is the case, for example, for the interaction between the carbon atoms of hexane and perfluorohexane, which is shown in Figure 4.

**Figure 4:**
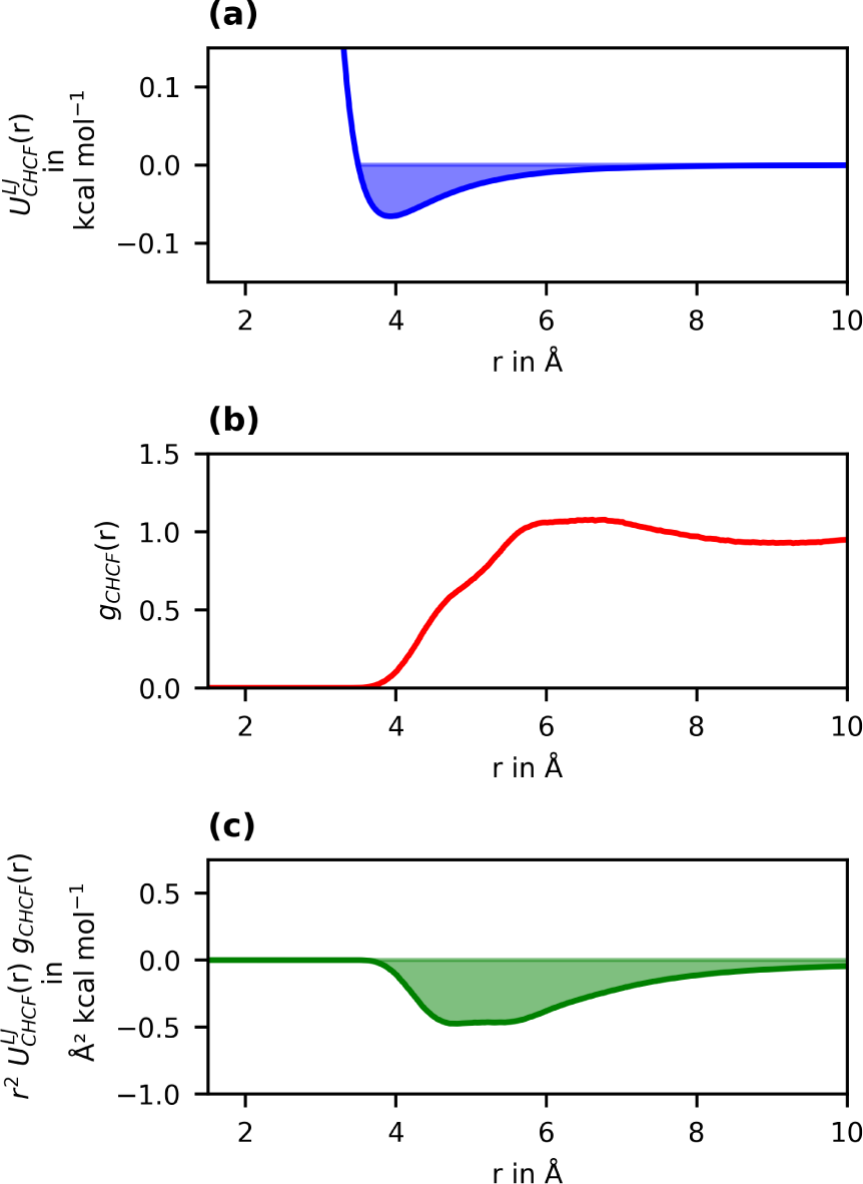
Elementary functions of the calculation of the effective interaction strength of the interaction between hexane and perfluorohexane carbon atoms. (a) C_H_–C_F_ interaction potential according to the OPLS-AA force field (12–6 Lennard-Jones, $\varepsilon_{C_{H}C_{F}}=0.066$ kcal/mol, $\sigma_{C_{H}C_{F}}=3.5$ Å).^11,39^ (b) Radial distribution function between hexane and perfluorohexane carbon atoms obtained from an MD simulation at 300 K. (c) *r* ^2^ *U*_*ab*_(*r*)*g*_*ab*_(*r*) as the integrand of eq 5.

The RDF *g*c_H_c_F_(*r*) shows that almost all atomic distances are larger than 4 Å, so that they are exclusively in the region where the interaction potential is attractive. Therefore, the integrand of eq 5 is zero up to distances of about 4 Å and then becomes negative. Long-range interactions again contribute to a large extent, as the RDF has a broad peak around 6.5 Å and decreases only slightly at even larger distances.

To provide a more complete picture, the absolute values of all pairwise effective interaction strengths are summarized in Table 1. In addition, Figure 5 visualizes how the individual interactions contribute to the molecular interaction energies $U_{C_{6}H_{14}-C_{6}H_{14}}^{\mathrm{eff}}$ , $U_{C_{6}F_{14}-C_{6}F_{14}}^{\mathrm{eff}}$ , and $U_{C_{6}H_{14}-C_{6}F_{14}}^{\mathrm{eff}}$ from eq 7, how the homo- and heteromolecular interactions differ, and how temperature affects their ratio and the effective energy of mixing. First, we note that the relative contribution of the pairwise interaction types to the effective homomolecular interaction energies differs between the molecule types. For hexane–hexane interactions, the C_H_–C_H_ interaction has the largest weight, and H–H interactions only play a minor role. The reverse is true for perfluorohexane–perfluorohexane interactions, where F–F interactions contribute the most, and C_F_–C_F_ interactions contribute the least. This significance of the contributions of C_H_ and F atoms is also reflected in the effective heteromolecular interaction energies, to which F–C_H_ interactions contribute almost twice as much as H–C_F_ interactions.

**Figure 5:**
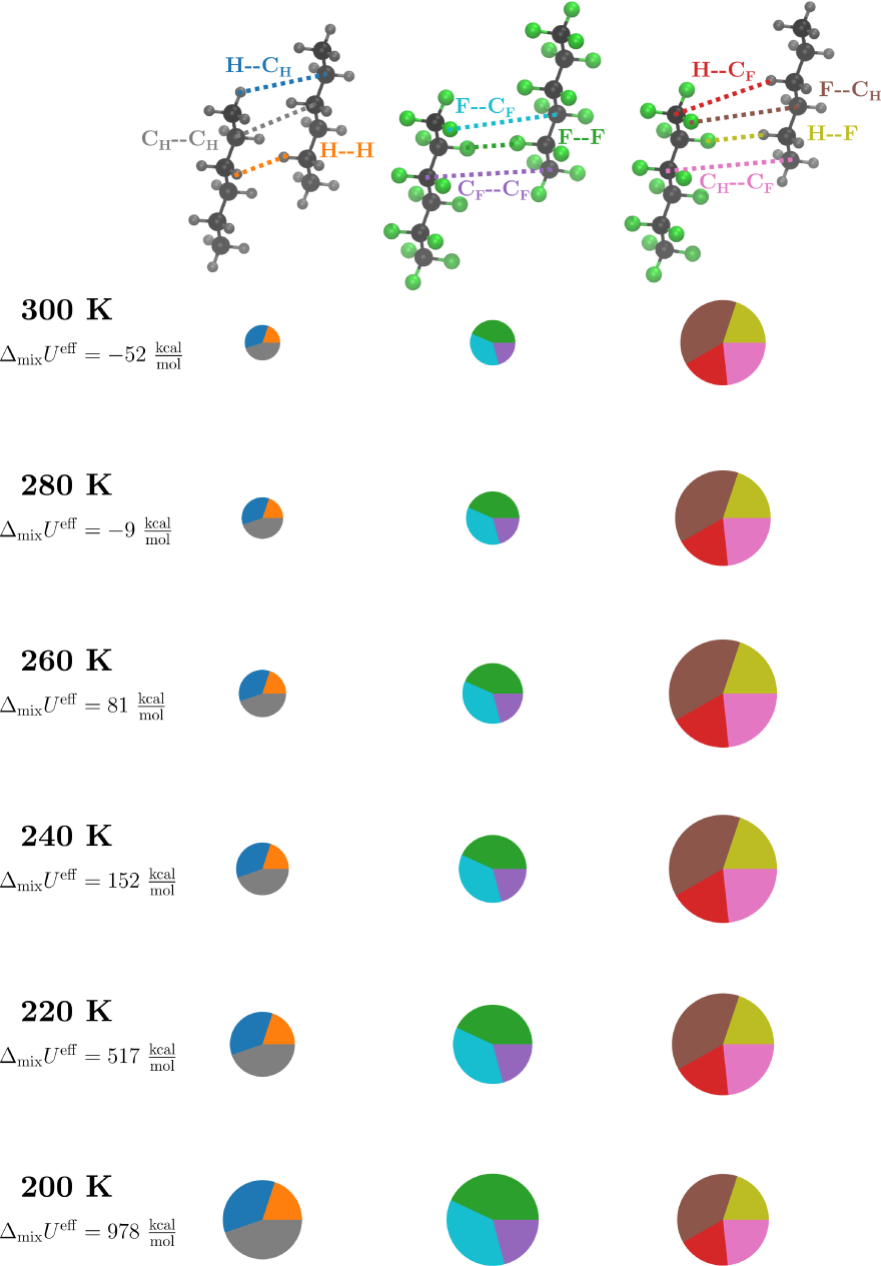
Effective interaction strengths of all pairwise interactions in a hexane–perfluorohexane mixture at temperatures between 200 and 300 K. The area of the circles is scaled according to the absolute values of $U_{C_{6}H_{14}-C_{6}H_{14}}^{\mathrm{eff}}$ , $U_{C_{6}F_{14}-C_{6}F_{14}}^{\mathrm{eff}}$ , and $U_{C_{6}H_{14}-C_{6}F_{14}}^{\mathrm{eff}}$ .

**Table 1:** Effective Interaction Strengths in kcal/mol of All Pairwise Interactions in a Hexane–Perfluorohexane Mixture Calculated from MD Simulations Using eq 5

*a*	*b*	*T* = 200 K	*T* = 220 K	*T* = 240 K	*T* = 260 K	*T* = 280 K	*T* = 300 K
F	F	–486	–419	–359	–322	–284	–243
F	C_F_	–408	–351	–300	–267	–234	–199
C_F_	C_F_	–236	–203	–173	–154	–135	–115
H	H	–193	–159	–129	–116	–101	–86
H	C_H_	–344	–281	–228	–205	–180	–154
C_H_	C_H_	–435	–356	–289	–260	–229	–197
H	F	–224	–248	–263	–247	–233	–208
F	C_H_	–430	–480	–508	–477	–451	–402
H	C_F_	–208	–232	–246	–230	–216	–192
C_H_	C_F_	–262	–292	–309	–289	–273	–243

The distribution of the relative contributions remains constant over all simulated temperatures, while the absolute values of $U_{C_{6}H_{14}-C_{6}H_{14}}^{\mathrm{eff}}$ , $U_{C_{6}F_{14}-C_{6}F_{14}}^{\mathrm{eff}}$ , $U_{C_{6}H_{14}-C_{6}F_{14}}^{\mathrm{eff}}$ , and Δ_mix_ *U* ^eff^ vary. With increasing temperature the effective homomolecular interaction energies $U_{C_{6}H_{14}-C_{6}H_{14}}^{\mathrm{eff}}$ and $U_{C_{6}F_{14}-C_{6}F_{14}}^{\mathrm{eff}}$ , as well as all individual effective interaction strengths contributing to them, become less negative. Interestingly, the individual effective interaction strengths between atoms of different molecule types and the absolute value of the effective heteromolecular interaction energy $U_{C_{6}H_{14}-C_{6}F_{14}}^{\mathrm{eff}}$ do not increase with temperature, as one would expect from the higher degree of mixing shown in the weaker homomolecular interactions and the increase in the peak height of the RDF between hexane and perfluorohexane atoms (Figure 2b). Instead, we observe a superposition with the thermal effect of stronger fluctuations reducing the effective interaction strength. Approximately, this thermal effect can be quantified by the formula for the entropy of an ideal mixture with the mole fractions *x*_*A*_ = *x*_*B*_ = 0.5. With *TR*Δ*S* = *TR*ln2 we obtain an increasing entropic contribution from 275.5 kcal/mol at 200 K to 413.2 kcal/mol at 300 K. For temperatures above 240 K, this entropic term progressively outweighs the enthalpic contributions, so that $U_{C_{6}H_{14}-C_{6}F_{14}}^{\mathrm{eff}}$ decreases with temperature. At lower temperatures, the enthalpic term dominates, so that $U_{C_{6}H_{14}-C_{6}F_{14}}^{\mathrm{eff}}$ increases between 200 and 240 K. Nonetheless, the effective energy of mixing Δ_mix_ *U* ^eff^ becomes more negative at higher temperatures, reflecting the higher degree of mixing. The transition from positive to negative values of Δ_mix_ *U* ^eff^ is between 260 and 280 K, whereas the RDFs show a transition from more separated to more mixed phases already at lower temperatures between 220 and 240 K.

## Conclusions

In this work, we propose the effective interaction strength, defined as a species-specific variant of the configurational internal energy of a molecular liquid, as a state-dependent enthalpy. This quantity is a measure of the atomic contributions of all relevant atom types to the actual intermolecular interaction energy. It can thus serve as a simple yet insightful tool to analyze the actual configurational state of a system in a given molecular simulation.

Complementary to the configurational entropy of mixing, which can be computed independently of any interaction energy,^52^ the effective interaction strength reflects the enthalpic part of the free enthalpy driving the mixing or demixing phase transition of molecular liquids. It can be used to explain the concept of a philicity match or mismatch of two compounds on the basis of the actual averaged interactions for each individual pair of atomic species. It is possible to follow the temperature dependence of the effective interaction strength values along a mixing/demixing phase transition in order to understand which type of interaction is responsible for a particular philicity of a compound.

In the specific case of normal and perfluoroalkane mixtures, we observe that the decisive competition of interactions around the mixing temperature occurs between the van der Waals forces of fluorine–fluorine versus fluorine–carbon species. As the mixing temperature is approached from below, the latter increase and compensate the otherwise dominating fluorine interactions.

## Supplementary Material



## Notes

Further details on MD simulations, and additional Tables S1–S4 and Figures S1–S8 describing the calculation of the effective interaction strengths, effective interaction energies, and effective energies of mixing (PDF)
