# Supplementary material for: Effective Interaction Strength in Simulations of Liquid Mixtures: A Configuration-Dependent Species-Specific Measure of Interaction Enthalpies
Source: J Phys Chem B. 2025 Jul 21;129(30):7818–25. doi: 10.1021/acs.jpcb.5c03339 (PMC12319905; doi:10.1021/acs.jpcb.5c03339)
Supplement: Supplementary file 1 [file jp5c03339_si_001.pdf]

Supporting Information: Effective Interaction Strength in Simulations of Liquid Mixtures: A Configuration-Dependent Species-Specific Measure of Interaction Enthalpies

Anna Luisa Upterworth and Daniel Sebastiani*

Institute of Chemistry, Martin Luther University Halle-Wittenberg, 06120 Halle, Germany

Molecular Dynamics Simulations

Table S1: Additional information regarding the composition of the simulation box at all simulated temperatures.

	T in K	V in Å^3	ρ in g/cm^3	$N_{C_6F_{14}}$	$N_{C_6H_{14}}$	N_F	N_{C_F}	N_H	N_{C_H}
1	200	129247.22	1.36	250	250	3500	1500	3500	1500
2	220	135242.69	1.30	250	250	3500	1500	3500	1500
3	240	142727.68	1.23	250	250	3500	1500	3500	1500
4	260	151589.95	1.16	250	250	3500	1500	3500	1500
5	280	162771.34	1.08	250	250	3500	1500	3500	1500
6	300	182766.92	0.96	250	250	3500	1500	3500	1500

Calculation of the Effective Interaction Strengths

Table S2: Integral values in Å^3 kcal mol^{-1} for all pairwise interactions in the hexane–perfluorohexane mixture obtained by integrating the integrand of Eq. (5) from the main text with the scipy.integrate.trapezoid method[S1] at all simulated temperatures.

a	b	T=200 K	T=220 K	T=240 K	T=260 K	T=280 K	T=300 K
F	F	-0.82	-0.74	-0.67	-0.63	-0.60	-0.58
F	C_F	-1.60	-1.44	-1.30	-1.23	-1.16	-1.10
C_F	C_F	-2.15	-1.94	-1.74	-1.65	-1.56	-1.49
H	H	-0.32	-0.28	-0.24	-0.23	-0.21	-0.20
H	C_H	-1.35	-1.15	-0.99	-0.94	-0.89	-0.85
C_H	C_H	-3.97	-3.41	-2.92	-2.79	-2.63	-2.54
H	F	-0.19	-0.22	-0.24	-0.24	-0.25	-0.25
F	C_H	-0.84	-0.98	-1.10	-1.09	-1.11	-1.11
H	C_F	-0.41	-0.48	-0.53	-0.53	-0.53	-0.53
C_H	C_F	-1.20	-1.40	-1.56	-1.55	-1.57	-1.57

Prefactors (Table S3) were calculated according to

$$4\pi \frac{N_a N_b}{2V} \tag{S1}$$

for interactions between atoms of the same molecule type, and according to

$$\left(4\pi \frac{N_a N_b}{V}\right) \tag{S2}$$

for interactions between atoms of different molecule types. Values used for the box volume V and the numbers of atoms of type a and b are given in Table S1.

Table S3: Prefactors in Å^{-3} resulting from Equations S1 and S2 for the calculation of the effective interaction strengths of pairwise interactions in the hexane–perfluorohexane mixture at all simulated temperatures.

a	b	T=200 K	T=220 K	T=240 K	T=260 K	T=280 K	T=300 K
F	F	595.52	569.12	539.27	507.74	472.87	421.13
F	C_F	255.22	243.91	231.12	217.60	202.66	180.49
C_F	C_F	109.38	104.53	99.05	93.26	86.85	77.35
H	H	595.52	569.12	539.27	507.74	472.87	421.13
H	C_H	255.22	243.91	231.12	217.60	202.66	180.49
C_H	C_H	109.38	104.53	99.05	93.26	86.85	77.35
H	F	1191.04	1138.24	1078.54	1015.49	945.73	842.26
F	C_H	510.44	487.82	462.23	435.21	405.31	360.97
H	C_F	510.44	487.82	462.23	435.21	405.31	360.97
C_H	C_F	218.76	209.06	198.10	186.52	173.71	154.70

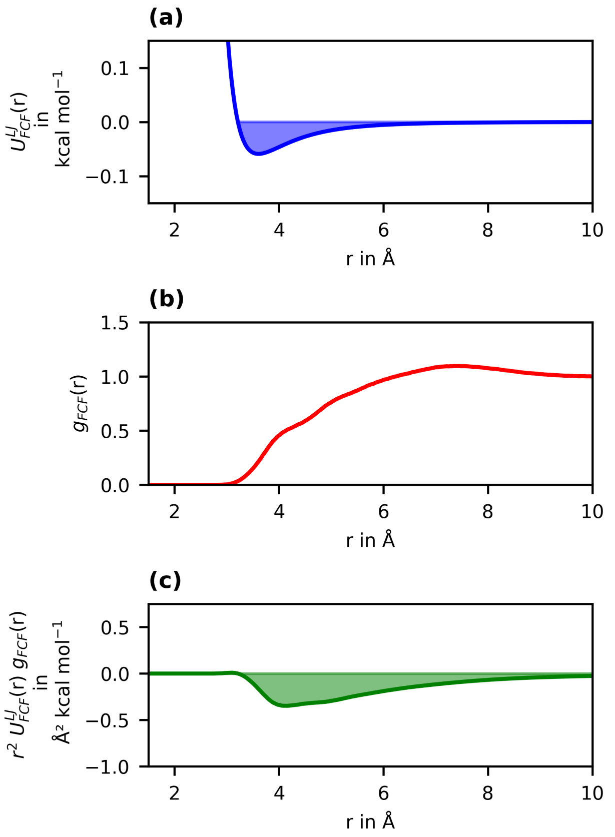

Figure S1: Elementary functions of the calculation of the effective interaction strength of the interaction between perfluorohexane fluorine and carbon atoms. (a) F–C_F interaction potential according to the OPLS-AA force field (12-6 Lennard-Jones, $\varepsilon_{FC_F} = 0.059\,\mathrm{kcal/mol}$, $\sigma_{FC_F} = 3.213\,\mathrm{\AA}$),[S2] (b) Radial distribution function between fluorine and perfluorohexane carbon atoms obtained from an MD simulation of a hexane–perfluorohexane mixture at 300 K. (c) $r^2 U_{FC_F}(r) g_{FC_F}(r)$ as the integrand of Eq. (5) from the main text.

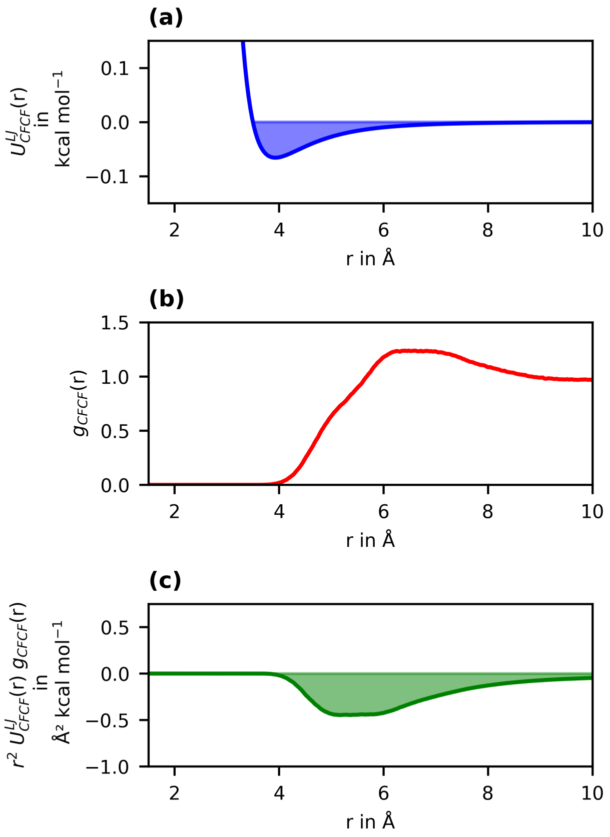

Figure S2: Elementary functions of the calculation of the effective interaction strength of the interaction between perfluorohexane carbon atoms. (a) C_F–C_F interaction potential according to the OPLS-AA force field (12-6 Lennard-Jones, $\varepsilon_{C_F C_F} = 0.066\,\mathrm{kcal/mol}$, $\sigma_{C_F C_F} = 3.5\,\text{Å}$),[S2] (b) Radial distribution function between perfluorohexane carbon atoms obtained from an MD simulation of a hexane–perfluorohexane mixture at 300 K. (c) $r^2 U_{C_F C_F}(r) g_{C_F C_F}(r)$ as the integrand of Eq. (5) from the main text.

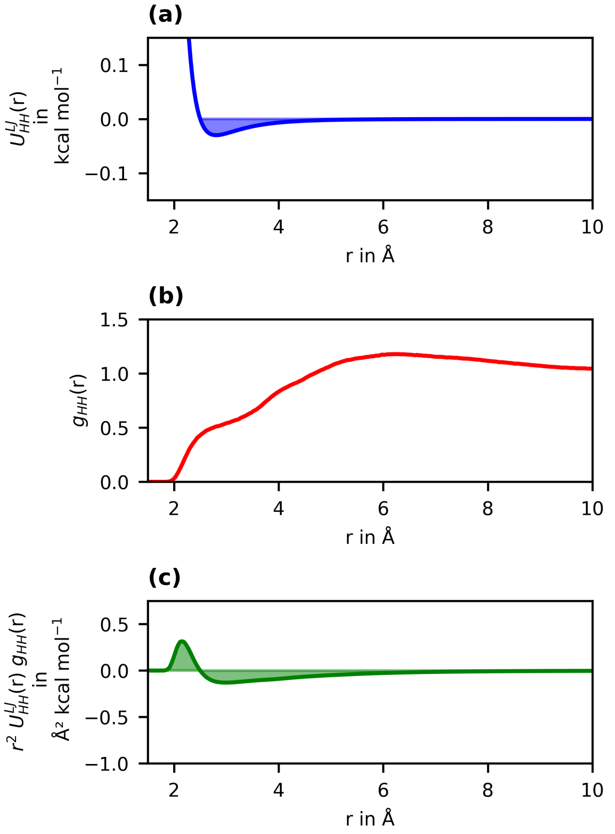

Figure S3: Elementary functions of the calculation of the effective interaction strength of the interaction between hexane hydrogen atoms. (a) H–H interaction potential according to the OPLS-AA force field (12-6 Lennard-Jones, $\varepsilon_{HH} = 0.03\,\mathrm{kcal/mol}$, $\sigma_{HH} = 2.5\,\text{Å}$),[S3] (b) Radial distribution function between hexane hydrogen atoms obtained from an MD simulation of a hexane–perfluorohexane mixture at 300 K. (c) $r^2 U_{HH}(r) g_{HH}(r)$ as the integrand of Eq. (5) from the main text.

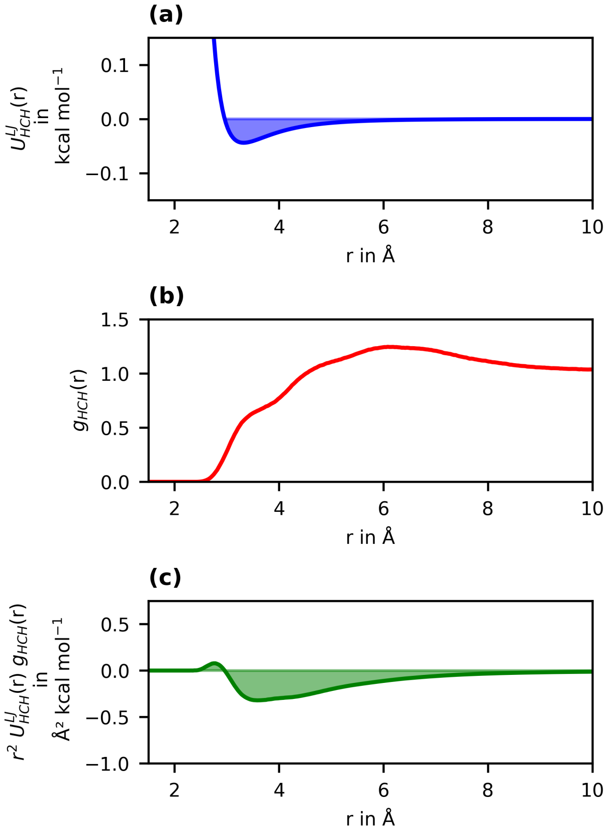

Figure S4: Elementary functions of the calculation of the effective interaction strength of the interaction between hexane hydrogen and carbon atoms. (a) H–C_H interaction potential according to the OPLS-AA force field (12-6 Lennard-Jones, $\varepsilon_{HC_H} = 0.044\,\mathrm{kcal/mol}$, $\sigma_{HC_H} = 2.96\,\mathrm{Å}$),[S3] (b) Radial distribution function between hexane hydrogen and carbon atoms obtained from an MD simulation of a hexane–perfluorohexane mixture at 300 K. (c) $r^2U_{HC_H}(r)g_{HC_H}(r)$ as the integrand of Eq. (5) from the main text.

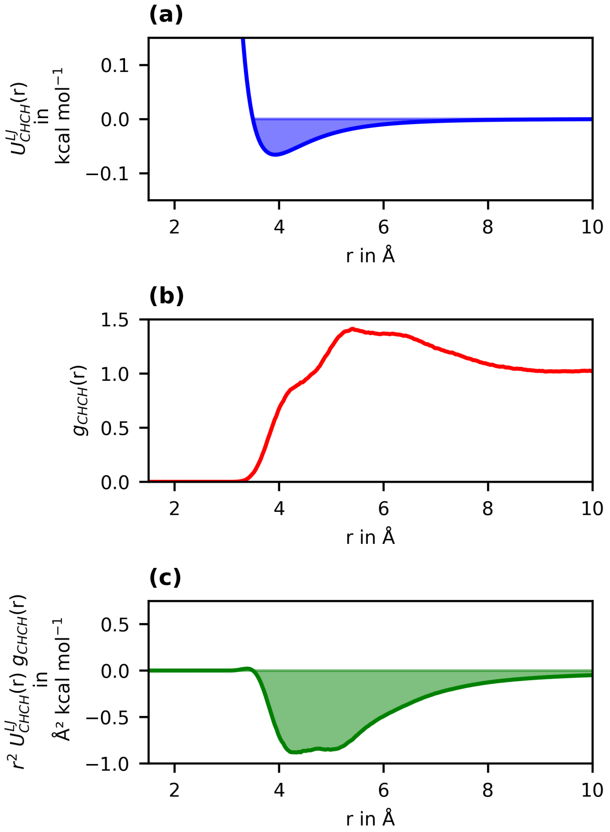

Figure S5: Elementary functions of the calculation of the effective interaction strength of the interaction between hexane carbon atoms. (a) C_H–C_H interaction potential according to the OPLS-AA force field (12-6 Lennard-Jones, $\varepsilon_{C_H C_H} = 0.066$ kcal/mol, $\sigma_{C_H C_H} = 3.5$ Å),[S3] (b) Radial distribution function between hexane carbon atoms obtained from an MD simulation of a hexane–perfluorohexane mixture at 300 K. (c) $r^2 U_{C_H C_H}(r) g_{C_H C_H}(r)$ as the integrand of Eq. (5) from the main text.

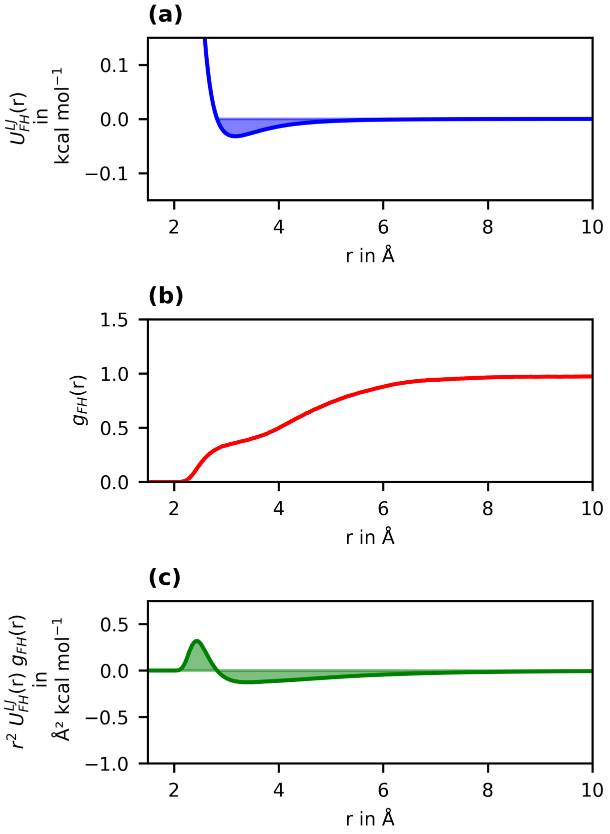

Figure S6: Elementary functions of the calculation of the effective interaction strength of the interaction between hydrogen and fluorine atoms. (a) H–F interaction potential according to the OPLS-AA force field (12-6 Lennard-Jones, $\varepsilon_{HF} = 0.032\,\text{kcal/mol}$, $\sigma_{HF} = 2.824\,\text{Å}$),[S2–S4] (b) Radial distribution function between hydrogen and fluorine atoms obtained from an MD simulation of a hexane–perfluorohexane mixture at 300 K. (c) $r^2 U_{HF}(r) g_{HF}(r)$ as the integrand of Eq. (5) from the main text.

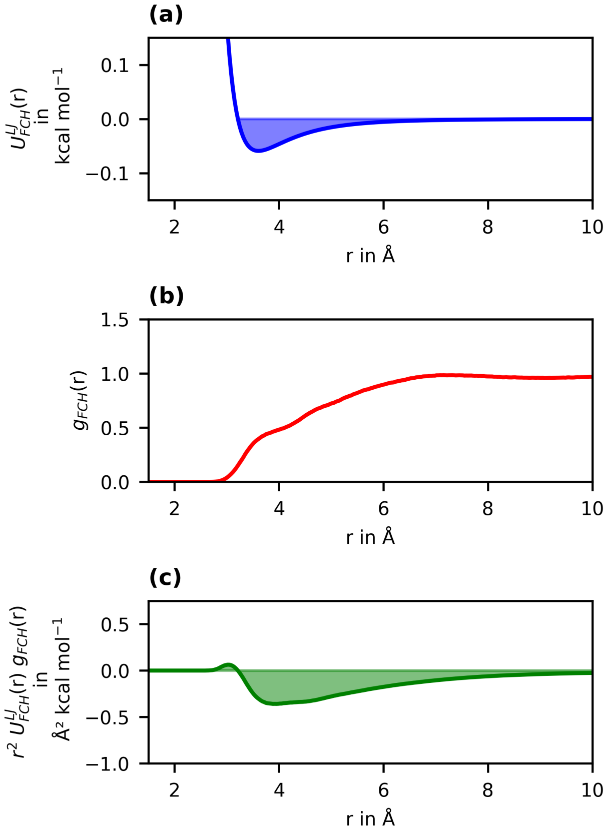

Figure S7: Elementary functions of the calculation of the effective interaction strength of the interaction between perfluorohexane fluorine and hexane carbon atoms. (a) F–C_H interaction potential according to the OPLS-AA force field (12-6 Lennard-Jones, $\varepsilon_{FC_H} = 0.059\,\mathrm{kcal/mol}$, $\sigma_{FC_H} = 3.213\,\mathrm{Å}$),[S2,S3] (b) Radial distribution function between fluorine and hexane carbon atoms obtained from an MD simulation of a hexane–perfluorohexane mixture at 300 K. (c) $r^2 U_{FC_H}(r) g_{FC_H}(r)$ as the integrand of Eq. (5) from the main text.

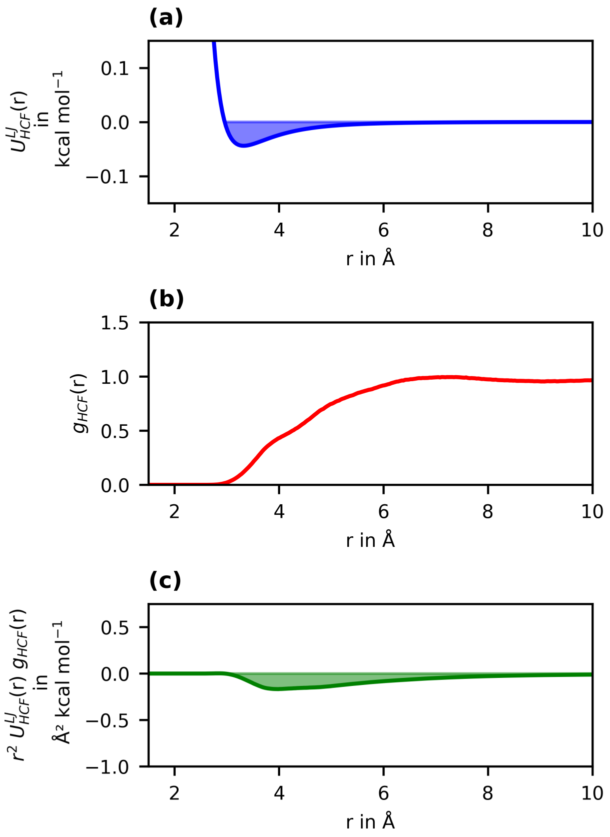

Figure S8: Elementary functions of the calculation of the effective interaction strength of the interaction between hexane hydrogen and perfluorohexane carbon atoms. (a) H–C_F interaction potential according to the OPLS-AA force field (12-6 Lennard-Jones, $\varepsilon_{HC_F} = 0.044\,\mathrm{kcal/mol}$, $\sigma_{HC_F} = 2.96\,\mathrm{Å}$),[S2,S3] (b) Radial distribution function between hydrogen and perfluorohexane carbon atoms obtained from an MD simulation of a hexane–perfluorohexane mixture at 300 K. (c) $r^2 U_{HC_F}(r) g_{HC_F}(r)$ as the integrand of Eq. (5) from the main text.

Effective interaction energies

Table S4: Effective interaction energies U^{eff}_{AB} and effective energy of mixing in kcal/mol at all simulated temperatures.

A	B	T=200 K	T=220 K	T=240 K	T=260 K	T=280 K	T=300 K
C_6F_{14}	C_6F_{14}	-1130	-973	-832	-744	-654	-557
C_6H_{14}	C_6H_{14}	-972	-797	-646	-581	-510	-437
C_6H_{14}	C_6F_{14}	-1124	-1252	-1326	-1242	-1174	-1045
$\Delta U^{\mathrm{eff}}_{\mathrm{mix}}$ in kcal/mol		-978	-518	-152	-80	10	51

References

(S1) Virtanen, P.; Gommers, R.; Oliphant, T. E.; Haberland, M.; Reddy, T.; Cournapeau, D.; Burovski, E.; Peterson, P.; Weckesser, W.; Bright, J.; van der Walt, S. J.; Brett, M.; Wilson, J.; Millman, K. J.; Mayorov, N.; Nelson, A. R. J.; Jones, E.; Kern, R.; Larson, E.; Carey, C. J.; Polat, u. l.; Feng, Y.; Moore, E. W.; VanderPlas, J.; Laxalde, D.; Perktold, J.; Cimrman, R.; Henriksen, I.; Quintero, E. A.; Harris, C. R.; Archibald, A. M.; Ribeiro, A. H.; Pedregosa, F.; van Mulbregt, P.; Vijaykumar, A.; Bardelli, A. P.; Rothberg, A.; Hilboll, A.; Kloeckner, A.; Scopatz, A.; Lee, A.; Rokem, A.; Woods, C. N.; Fulton, C.; Masson, C.; Häggström, C.; Fitzgerald, C.; Nicholson, D. A.; Hagen, D. R.; Pasechnik, D. V.; Olivetti, E.; Martin, E.; Wieser, E.; Silva, F.; Lenders, F.; Wilhelm, F.; Young, G.; Price, G. A.; Ingold, G.-L.; Allen, G. E.; Lee, G. R.; Audren, H.; Probst, I.; Dietrich, J. P.; Silterra, J.; Webber, J. T.; Slavič, J.; Nothman, J.; Buchner, J.; Kulick, J.; Schönberger, J. L.; de Miranda Cardoso, J. V.; Reimer, J.; Harrington, J.; Rodríguez, J. L. C.; Nunez-Iglesias, J.; Kuczynski, J.; Tritz, K.; Thoma, M.; Newville, M.; Kümmerer, M.; Bolingbroke, M.; Tartre, M.; Pak, M.; Smith, N. J.; Nowaczyk, N.; Shebanov, N.; Pavlyk, O.; Brodtkorb, P. A.; Lee, P.; McGibbon, R. T.; Feldbauer, R.; Lewis, S.; Tygier, S.; Sievert, S.; Vigna, S.; Peterson, S.; More, S.; Pudlik, T.; Oshima, T.; Pingel, T. J.; Robitaille, T. P.; Spura, T.;

Jones, T. R.; Cera, T.; Leslie, T.; Zito, T.; Krauss, T.; Upadhyay, U.; Halchenko, Y. O.; Vázquez-Baeza, Y.; Contributors, S. . SciPy 1.0: fundamental algorithms for scientific computing in Python. *Nat. Methods* **2020**, *17*, 261–272.

(S2) Watkins, E. K.; Jorgensen, W. L. Perfluoroalkanes: Conformational Analysis and Liquid-State Properties from ab Initio and Monte Carlo Calculations. *J. Phys. Chem. A* **2001**, *105*, 4118–4125.

(S3) Jorgensen, W. L.; Maxwell, D. S.; Tirado-Rives, J. Development and Testing of the OPLS All-Atom Force Field on Conformational Energetics and Properties of Organic Liquids. *J. Am. Chem. Soc.* **1996**, *118*, 11225–11236.

(S4) Morgado, P.; Martins, L. F. G.; Filipe, E. J. M. From nano-emulsions to phase separation: evidence of nano-segregation in (alkane + perfluoroalkane) mixtures using 129Xe NMR Spectroscopy. *Phys. Chem. Chem. Phys.* **2019**, *21*, 3742–3751.
